# Monitoring the thin film formation during sputter deposition of vanadium carbide

**DOI:** 10.1107/S1600577514024412

**Published:** 2015-01-01

**Authors:** Marthe Kaufholz, Bärbel Krause, Sunil Kotapati, Martin Köhl, Miguel F. Mantilla, Michael Stüber, Sven Ulrich, Reinhard Schneider, Dagmar Gerthsen, Tilo Baumbach

**Affiliations:** a ANKA/Institut für Photonenforschung und Synchrotronstrahlung, Karlsruher Institut für Technologie, Karlsruhe, Germany; b Max-Planck-Institut für Intelligente Systeme, Stuttgart, Germany; c Institut für Angewandte Materialien – Angewandte Werkstoffphysik, Karlsruher Institut für Technologie, Karlsruhe, Germany; d Laboratorium für Elektronenmikroskopie, Karlsruher Institut für Technologie, Karlsruhe, Germany

**Keywords:** *in situ* X-ray reflectivity, composite, sputter deposition

## Abstract

The thin film formation of magnetron sputtered polycrystalline coatings was monitored by *in situ* X-ray reflectivity measurements. The measured intensity was analyzed using the Parratt algorithm for time-dependent thin film systems. Guidelines for the on-line interpretation of the data were developed. For thick coatings, the experimental resolution needs to be included in the data evaluation in order to avoid misinterpretations. Based on a simple layer model, the time-dependent mean electron density, roughness and growth velocity were extracted from the data. As an example, the method was applied to the hard coating material vanadium carbide. Both instantaneous and slowly varying changes of the coating could be detected. It was shown that the growth velocity is proportional to the DC power. Significant changes of the microstructure induced by the working gas pressure are mainly driven by the chemical composition.

## Abstract 2

The theoretical description and the experimental realisation of *in situ* X-ray reflectivity measurements during thin film deposition of polycrystalline vanadium carbide coatings are presented.

## 1. Introduction   

Magnetron sputtered polycrystalline coatings are widely used, for example as protective hard coatings for cutting tools and medical implants, or as anti-reflection coatings for solar cells and glasses. The deposition conditions during the sputter process affect different aspects of the microstructure such as roughness, texture and grain size of the film. These, in turn, influence the optical and mechanical properties of the coating. However, the relation between deposition conditions and microstructure formation is not yet fully understood. Monitoring the material distribution during growth, using a non-destructive probe compatible with the high-pressure conditions, can give valuable information about the thin film formation. *In situ* X-ray scattering techniques fulfill these conditions. For epitaxial systems, measurements at fixed points of a crystal truncation rod have been successfully performed by Braun *et al.* (2003 ▶) ▶, Krause *et al.* (2004 ▶) ▶, Jenichen *et al.* (2007 ▶) ▶, Woll *et al.* (2011 ▶) ▶ and Chinta & Headrick (2014 ▶) ▶. However, this method is not applicable for polycrystalline coatings. *In situ* small-angle X-ray scattering methods such as, for example, GISAXS or *in situ* X-ray reflectivity (XRR) measurements are independent of the crystalline structure and can be applied to almost any coating (Renaud *et al.*, 2009 ▶; Yu *et al.*, 2013 ▶). For *ex situ* samples, XRR is a well established method used by a large community (Tolan, 1999 ▶). *In situ* XRR measurements were carried out using energy-dispersive reflectivity setups (Kowarik *et al.*, 2007 ▶) or monochromatic radiation. In the latter case, *in situ* XRR measurements can be performed by *scanning* the angular range (Chiarello *et al.*, 1997 ▶) or measuring at a fixed angular position (Louis *et al.*, 1994 ▶; Peverini *et al.*, 2005 ▶; Lee *et al.*, 2008 ▶).

In this manuscript we focus on *in situ* measurements using monochromatic X-rays (§2). Different measurement types are compared (§3). A theoretical approach for the quantitative and qualitative analysis of the *in situ* data is developed (§4). This approach is applied to polycrystalline hard coating materials, which usually have a coating thickness in the micrometre regime. Hence the industrial sputter process typically involves high deposition rates (nm s^−1^) and long deposition times (up to hours). It will be shown that the experimental resolution significantly affects the measured data of thick coatings. However, taking this into account it is still possible to monitor the temporal changes of the thickness, roughness and electron density of the film. This allows one to gain insight into the instant response of the microstructure to growth parameter changes as occurring, for example, during the deposition of complex gradient samples.

As a model system, the growth of the composite material vanadium carbide (VC) was studied (§5). The thin films were deposited on silicon by DC sputtering from a compound target. Similarly to TiC, VC can form two separate phases during the deposition, VC_1–*x*_ in cubic rock salt structure and amorphous carbon (a-C) (Pflüger *et al.*, 1984 ▶; Stüber *et al.*, 2002 ▶; El Mel *et al.*, 2010 ▶). This phase composition is important for the mechanical properties of the coating, but depends strongly on the growth conditions (Liao *et al.*, 2005 ▶; Eklund *et al.*, 2007 ▶; Neidhardt *et al.*, 2008 ▶; Zhang *et al.*, 2013 ▶). Two important growth parameters are DC power (§5.1) and working gas pressure (§5.2). We will show that the DC power affects mainly the deposition rate, while the working gas pressure has a strong influence on the composition and thus on the microstructure formation.

## 2. Experimental and simulation details   

In the following the sample preparation and the experimental details of the *in situ* measurements are described. The details of the complementary measurements and the simulation of the sputter process are presented.

### 2.1. Sample preparation   

**Figure 1:**
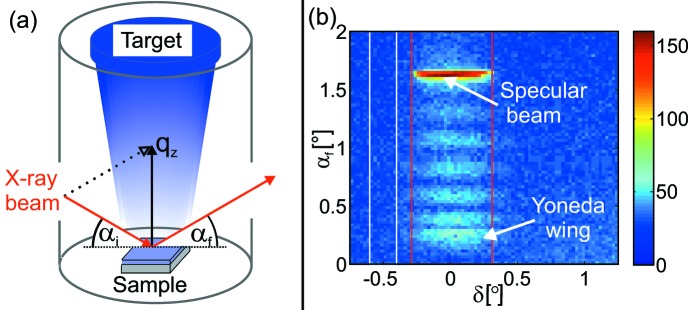
(*a*) Scheme of the experimental setup. The X-ray measurements are performed during the deposition process. The X-ray reflectivity geometry is indicated, showing the notation of 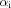, 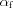 and 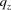 as they are used in the text. (*b*) PILATUS image acquired during the *stationary* measurement at 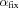 = 1.6° at 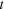 = 180 s of Lp-a. The integration ranges for the specular beam and the diffuse are indicated by boxes. The Yoneda wing is indicated by an arrow (see §5.2).

Vanadium carbide (VC_1–*x*_/a-C) thin films were deposited on silicon by DC magnetron sputtering using the *in situ* sputtering system described by Krause *et al.* (2012 ▶). Fig. 1(*a*) ▶ shows schematically the sputter geometry. The nominally stoichiometric VC compound target (MaTecK GmbH) with a 3 inch diameter was mounted at the top of the chamber. The distance between target and substrate was 12 cm. The silicon (100) substrates had a lateral size of 20 mm × 20 mm and a height of 0.5 mm. The substrates were covered by a natural oxide layer with an average thickness of about 3 nm, which was verified by X-ray reflectivity and TEM measurements.

All thin films presented in this paper were grown at room temperature. The base pressure of the system was 10^−6^ Pa. Argon was used as sputtering gas. For the comparison of two *in situ* measurement types (§3), two samples (Lp-a and Lp-b) were deposited under the following growth conditions. A high Ar flux of 10 sccm was required for the plasma ignition at a DC power of 50 W. The high flux resulted in an increase in the chamber pressure up to *p* = 4 Pa. After 10 s, the Ar flux was reduced to 0.4 sccm. The working gas pressure decreased to *p* = 0.2 Pa. Simultaneously, the power was ramped to the final value of 200 W within 20 s, to avoid cracking of the target. These conditions were kept constant for 600 s. Then the DC power was ramped down to 50 W, before the growth was stopped. The typical development of pressure and DC power is presented in Fig. 7(*c*).

For the investigation of the influence of the DC power on the thin film formation, the sample P-step was grown (§5.1). After the plasma ignition, the DC power was increased stepwise every 250 s by 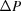 = 25 W from 50 W to 200 W.

For the investigation of the influence of the pressure on the thin film formation (§5.2), the sample HP-a was grown at a constant pressure of *p* = 4 Pa. The total deposition time including ramping was *t* = 650 s. The development of the main process parameters is presented in Fig. 7(*a*).

For complementary measurements [X-ray diffraction (XRD) and X-ray photoemission spectroscopy], Lp-c and HP-b were deposited under the same growth conditions as Lp-a and HP-a, respectively.

### 2.2. *In situ* measurements   

*In situ* XRR measurements during sputter deposition of VC were performed at the MPI beamline at ANKA (Stierle *et al.*, 2004 ▶). The *in situ* sputtering system was mounted on a HUBER 4+2 circle diffractometer. Measurements were performed using monochromatic X-rays with an energy *E* = 10 keV (λ = 1.24 Å) and a beam size of 300 µm × 300 µm (horizontal × vertical) focused on the sample.

For monochromatic radiation, two measurement types using the same geometry can be used to monitor the specular beam during the growth (Fig. 1*a* ▶):

(i) *Scanning* measurements. A *standard* XRR measurement is performed. The incident angle 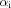 is scanned. The specularly reflected beam with 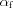 = 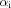 is measured. The *z*-component of the scattering vector 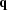 is related to 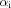 and 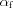 *via* 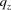 = 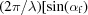 + 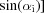. The scan is repeated until, for example, the deposition of a film is finished.

(ii) *Stationary* measurements at a fixed angular position. The specular intensity is monitored at a fixed angular position 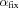 = 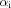 = 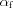, *i.e.* 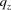 is constant during the measurement.

Examples for *scanning* and *stationary* measurement types will be shown in §3.

The *scanning* measurements were performed with a scan range of 0.1° to 4.1° and an angular step size of 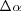 = 0.005°, using a NaI scintillator with automatic absorbers. For the *stationary* measurements at a fixed angle 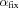 = 1.6°, a PILATUS 100 K detector with a pixel size of 172 µm was used. The images with integration time of 1 s were taken in time steps of 1.1 s. Fig. 1(*b*) ▶ shows a PILATUS image during the deposition of Lp-a at *t* = 180 s. δ corresponds to the angle in the horizontal plane relative to the direct beam. Besides the specular beam, the two-dimensional detector additionally recorded the diffuse scattering. For each time-step, the intensity of the specular beam was integrated (including the diffuse scattering close to the specular beam due to roughness) and the average scattering background of the sample environment subtracted. The error due to the diffuse scattering is smaller than 0.01% and can therefore be neglected.

The stability of the setup was verified by the PILATUS images. No drifts of the specular beam were detected during the deposition.

### 2.3. Complementary methods   

High-resolution transmission electron microscopy (HRTEM) images of the sample P-step were taken with an aberration-corrected FEI Titan^3^ 80–300 microscope operated at 300 keV accelerating voltage, using a 4 Mpixel CCD camera (Gatan UltraScan 1000 P). The exposure time was 0.5 s, the information limit was 0.08 nm. For preparation of the cross-section sample, two pieces of P-step were glued face-to-face. After mechanical thinning of the 305 µm-thick disks, electron-transparent areas were obtained by Ar^+^ ion thinning at 3 kV in a Gatan precision ion polishing system (PIPS model 691).

X-ray photoemission spectroscopy (XPS) of Lp-a and HP-a were performed at the UHV Analysis Laboratory at ANKA. The sputter deposition chamber as well as the XPS analysis chamber are connected *via* a transfer system, allowing XPS measurements without exposing the samples to ambient conditions. For the XPS measurements, a Phoibos 150 analyzer and an unmonochromated XR-50 Mg *K*α X-ray source from SPECS were used [for a detailed description see Krause *et al.* (2013 ▶)]. The base pressure of the XPS chamber was 1 × 10^−8^ Pa. No beam-induced changes of the spectra were detected. The XPS spectra were analyzed with the software *CasaXPS* (Fairley & Carrick, 2005 ▶).

Specular XRD measurements of Lp-a and HP-a were performed in parallel beam geometry, using a Rigaku Smart­Lab diffractometer with Cu *K*α radiation (λ = 1.542 Å). The Cu *K*β lines were suppressed by a Ni filter.

### 2.4. Simulations of the sputter process   

Simulations of the sputter process from a compound target were performed following Mahieu *et al.* (2006 ▶) and Neidhardt *et al.* (2008 ▶). The initial energetic and angular distributions of the sputtered particles at the target position were simulated using *TRIDYN* (Möller *et al.*, 1988 ▶). *TRIDYN* covers the dynamic interaction of the individual elements in the compound target. The energy of the impinging Ar atoms of 300 eV was taken equal to 75% of the discharge voltage of 400 V (Fillon *et al.*, 2010 ▶). The *TRIDYN* results, the experimental conditions and the sputter chamber geometry described in §5.2 were used as simulation input for the software tool *SIMTRA* (Van Aeken *et al.*, 2008 ▶). *SIMTRA* calculates the trajectories of the sputtered particles in the gas phase. The simulations were performed independently for C and V. This approximation is valid since the sputtered atoms collide mostly with the Ar atoms.

## 3. Measurement methods during sputter deposition   

In this section, the two *in situ* measurement types during the sputter deposition are compared. Experimental data were collected under identical growth conditions.

**Figure 2:**
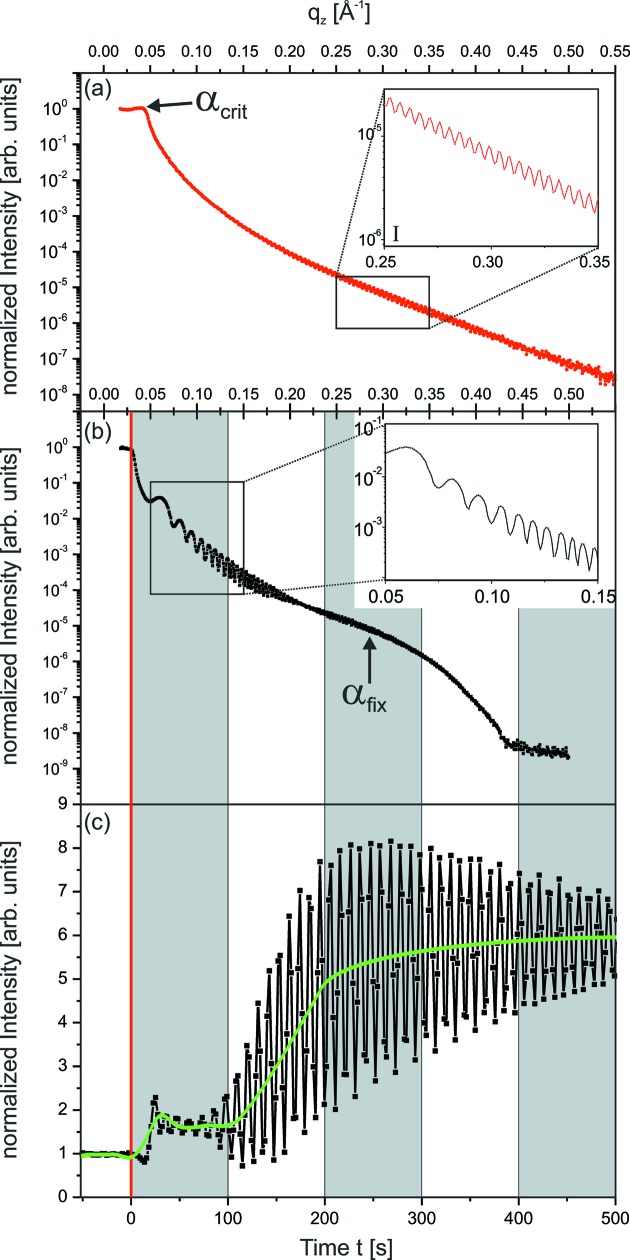
(*a*) *Scanning* XRR measurement of Lp-a after the deposition of 600 s. (*b*) *Scanning* measurement of Lp-b during the first 400 s of deposition and (*c*) *stationary* measurement of Lp-a during the first 500 s of deposition. The mean value of the intensity is marked by the green line. The time axis corresponds to both curves. The grey boxes indicate time intervals of 100 s. At *t* = 0 s (red line) the plasma was ignited. The insets present zooms of highlighted regions for better visualization.

As a reminder, Fig. 2(*a*) ▶ shows a *standard* XRR measurement of Lp-a performed after growth as an example of a typical *scanning* measurement of a thin film. The critical angle 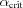, at which total external reflection occurs, is related to the electron density (Pietsch *et al.*, 2004 ▶). In the case of Lp-a, 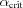 = 0.25° [denoted by an arrow in Fig. 2(*a*) ▶] corresponds to an electron density of 88% of the theoretical bulk density of VC_1–*x*_/a-C. The thickness oscillations (called the Kiessig fringes) arise due to the interference of the X-rays which are reflected at the surface and at the interface between coating and the substrate (Kiessig, 1931 ▶). The Kiessig fringes are highlighted in the inset in Fig. 2(*a*) ▶. The period 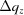 of the Kiessig fringes is related to the thickness *D* of the thin film by *D* = 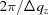.

Figs. 2(*b*) and 2(*c*) ▶ show experimental data of both *in situ* measurement types during the first 400 s of VC deposition. The *in situ* *scanning* measurement of Lp-b is presented in Fig. 2(*b*) ▶. The scan was performed from small to large 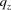 values. During the deposition, the coating thickness increases while scanning 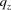 towards larger values. The deposition process started at 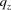 ≃ 0.025 Å^−1^, marked by the red line.

The curve exhibits the typical features of a standard XRR measurement. The critical angle is located at 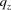 ≃ 0.031 Å^−1^. This is in good agreement with the expected value for the Si substrate since only a low amount of material was deposited at 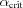. As highlighted in Fig. 2(*b*) ▶ (inset), the period of the Kiessig fringes decreases with increasing 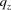, *i.e.* increasing film thickness. Therefore, for a given sampling frequency, at large coating thicknesses the oscillation period cannot be reliably determined.

Fig. 2(*c*) ▶ shows the *stationary* measurement at a fixed angular position of 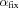 = 1.6° of Lp-a. The corresponding 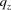-value is indicated by an arrow in Fig. 2(*b*) ▶. Growth oscillations are well resolved during the entire deposition time. The period varies only slightly, while the amplitude and the mean value of the intensity (green line) show significant time-dependent changes.

For optimization of the *scanning* *in situ* measurements, the distance of the data points needs to be constantly adapted to resolve the growth oscillations. This is not necessary for *stationary* measurements. Here, the growth oscillations are well resolved, even for larger coating thicknesses. Hence, *stationary* measurements have advantages in the case of polycrystalline thin films deposited with high deposition rates and long deposition times.

For a detailed understanding of *stationary* measurements, simulations were performed. They will be discussed in the next section.

## 4. Simulation of the *in situ* measurements   

### 4.1. Parratt algorithm for growing films   

**Figure 3:**
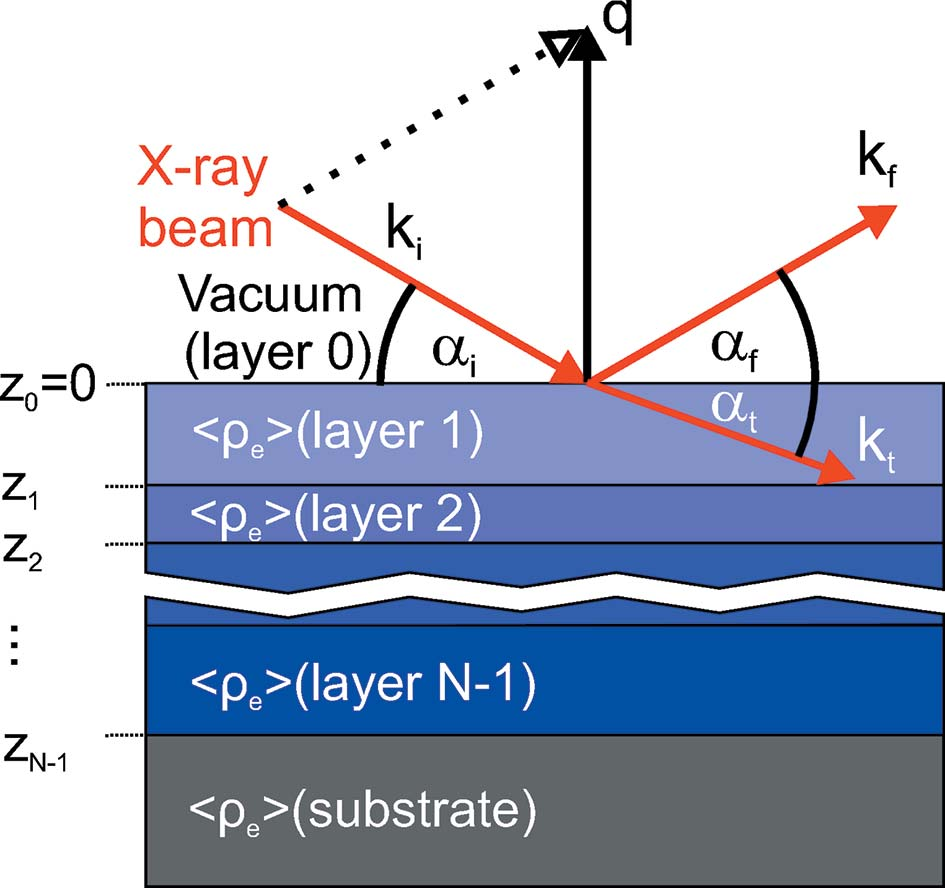
Scheme of the X-ray reflectivity geometry, showing the notation of 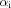, 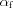, 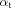, 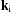, 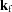, 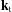, 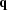 as they are used in the text. The sample is sliced into a virtual stack of layers *j* with height 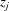. This virtual stack is used for the calculation of the XRR curve. For further details see text.

The Parratt algorithm is widely used for the simulation and fitting of *scanning* XRR curves of thin films (Parratt, 1954 ▶). In the following we summarize the Parratt algorithm, taking into account the time-dependence of the parameters which are needed for the description of the *in situ* XRR curves of a growing film. The thin film is described by an electron density profile 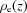. For applying the Parratt algorithm, 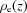 is sliced into a stack of *j* = 0, 1,…, *N* layers. This is schematically shown in Fig. 3 ▶. Each layer has a mean electron density 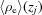 where 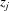 is the distance of the layer *j* to the sample surface at 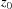 = 0. 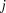 = 0 corresponds to the layer above the sample surface.

During the *in situ* experiment the electron density profile 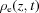 of the growing film changes with deposition time. 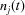 is the time-dependent refractive index of the layer *j*, which is related to the mean electron density 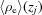 of the layer (Pietsch *et al.*, 2004 ▶). Therefore, the *z*-component of the wavevector, 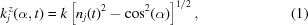
with *k* = 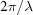, is also time-dependent. Note that, in the case of *scanning measurements*, α is changing in time too.


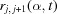
 and 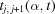 are the time-dependent Fresnel coefficients (here for S-polarization): 
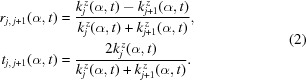
Hence, during growth of the sample, the reflectance 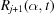 = 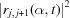 and transmission 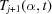 = 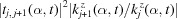 for each interface at 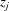 change with time.

The ratio 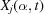 of the reflected and the transmitted X-ray beam at each interface is derived recursively by
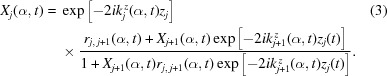
Since the substrate is assumed to be semi-infinite, no X-ray is reflected at the bottom of the sample, *i.e.* 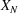 = 0. The specular intensity 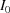 is calculated by 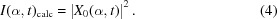
In the case of rough films, the diffuse scattering from the sample leads to a decrease in the specular intensity *I*. For small roughnesses, the effective Fresnel coefficient 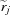 is used (Nevot & Croce, 1980 ▶; Sinha *et al.*, 1988 ▶): 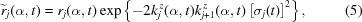
where 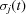 is the time-dependent r.m.s.-roughness of the layer *j*.

These formulae describe the *scanning* as well as *stationary* measurement types. In the following we focus on the *stationary* measurements, since these are the preferred measurement types in the case of polycrystalline coatings as discussed in §3.

### 4.2. Calculation of the rate of the thickness increase   

As presented in Fig. 2(*c*) ▶, growth oscillations are one characteristic feature of *stationary* measurements. In this section, we show how the period of these oscillations is related to the rate of thickness increase, 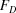, of the thin film. For that, we assume a simple one-layer system (*N* = 2). A VC_1–*x*_/a-C film is deposited on a Si substrate with 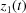 = 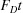. For simplicity, time-dependent changes in roughness and electron density are neglected. In this case 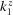 is constant. Equation (3) simplifies to 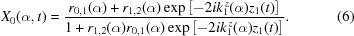
Only the exponent is time-dependent. The specular intensity *I* is calculated *via* equation (4). For 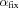 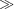 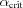, the intensity can be approximated by the first order of the Fourier series of *I*: 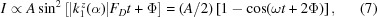
where 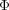 = π/2 if the thin film has a higher optical density than the substrate and 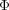 = 0 in the opposite case. The amplitude *A* is approximately constant for 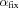 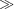 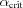.

This means that the specular intensity oscillates during the deposition. The oscillation period 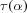 = 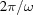 can be easily determined from the experimental data. The frequency 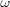 = 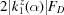 is only influenced by 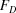, since 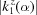 is constant at a fixed angular position. 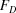 can be calculated from the oscillation period τ by
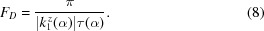
Equation (8) is not only valid for the here-discussed model system but also for more complex systems. For 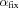 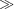 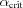, the oscillation period is almost independent of roughness changes. Even large density variations have only a slight influence on τ. Furthermore, this formula can be used for multilayer systems, as long as only the topmost layer is changing.

Hence in most of the cases, the thickness increase of the thin film can be easily determined during the deposition process and can be used as a direct feedback signal for the experimentalist. The thickness increase of the thin film is not necessarily equivalent to the deposition rate. At the same deposition rate, a porous film is growing faster in thickness than a film without voids.

### 4.3. Influence of the experimental setup   

**Figure 4:**
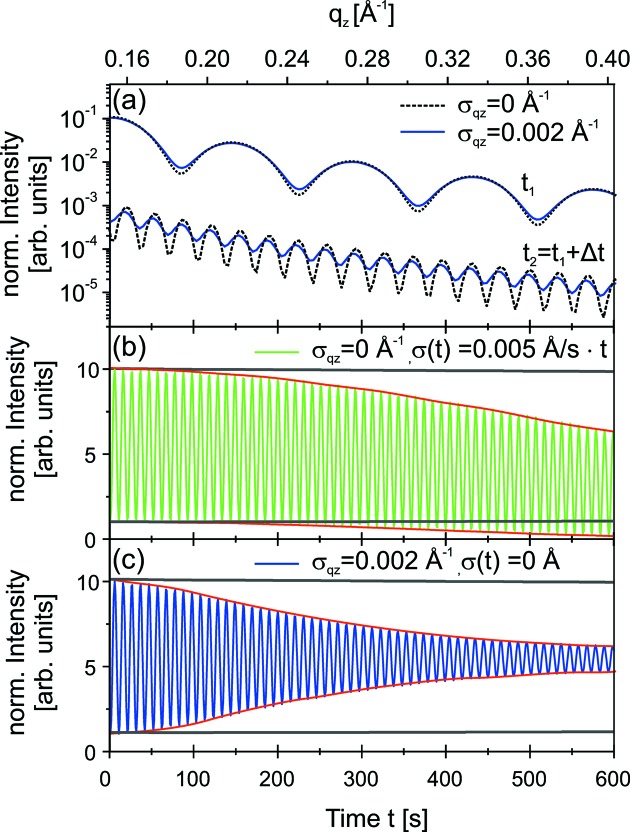
(*a*) Simulation of *scanning* XRR curves after the deposition at 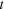 = 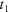 and 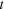 = 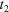, using the resolution elements 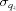 = 0 Å^−1^ (dotted lines) and 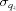 = 0.002 Å^−1^ (continuous lines). The upper curve is shifted for a better comparison. At 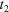 = 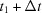, the influence due to 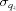 increases. (*b*), (*c*) Simulation of *stationary* XRR curves at 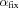 = 1.6° for a deposition time 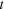 = 600 s with (*b*) 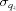 = 0 Å^−1^ and a linear increase in roughness [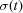 = *t* × 0.005 Å/s], and (*c*) including only a finite resolution (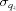 = 0.002 Å^−1^, σ = 0 Å). The envelopes of all curves are shown as red lines. The envelope of the simulated curve with 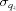 = 0 Å^−1^ and σ = 0 Å is presented by grey lines in (*b*) and (*c*) for comparison.

In this section, we show how the experimental resolution influences the amplitude. The calculations in §4 assumed a parallel X-ray beam. In reality, the X-ray beam is divergent. 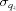 is the so-called *resolution element*. It describes the area in reciprocal space which is illuminated by the X-ray beam and accepted by the detector (Pietsch *et al.*, 2004 ▶). The smearing of a *standard* XRR curve with the specular intensity 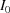 due to 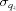 can be described by the resolution function 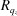 using a Gaussian function (Pedersen & Hamley, 1994 ▶),
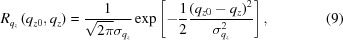
where 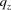 = 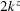 is the scattering vector of the reflectivity curve and 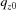 the nominal setting of the instrument. The measured reflectivity 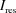 is given by 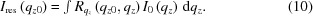
Fig. 4(*a*) ▶ shows the calculated XRR curves for two deposition times 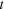 = 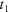 and 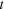 = 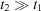. The black dotted curves correspond to 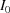 (no resolution element), the blue curves to 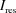, considering a resolution element of 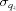 = 0.002 Å^−1^. The period of the Kiessig fringes at 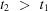 is shorter due to the larger film thickness. Deviations from 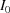 due to 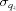 = 0.002 Å^−1^ are larger for the film with larger thickness. Fig. 4 ▶ presents two calculated *stationary* curves with (*b*) 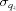 = 0 Å^−1^ and including only a linear increase in roughness from 0 Å to 3 Å [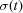 = *t* × 0.005 Å/s], and (*c*) including only a finite resolution [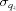 = 0.002 Å^−1^, 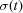 = 0 Å]. For comparison, the simulated envelope of the *stationary* curve with 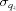 = 0 Å^−1^ and 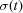 = 0 Å is presented (grey dotted line). Here σ is the roughness of the VC film. The roughness of the substrate was kept unchanged at 0 Å. The simulation parameters correspond to our experimental values.

The envelope of the *stationary* curve is almost constant. The roughness of the film leads to a slight decrease of the amplitude with time, while the resolution leads to a much stronger damping of the oscillations. The reduction of the intensity 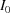 due to the resolution element can be described by
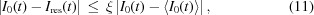
where 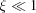 is the parameter quantifying the tolerated deviation and 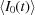 is the mean value of 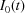.

In the following, we will derive a rule of thumb to estimate a certain critical thickness 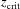 at which the maximal deviation ξ is reached. We use the simplified expression of 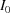 [equation (7)] for solving the integral in equation (10): 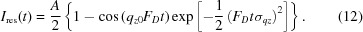
The exponential function describes the modification of the envelope of the intensity due to the resolution. Inserting equation (12) into equation (11) yields 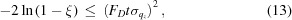
where 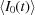 = *A*/2. Using 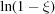 ≃ 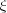 for 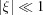, the following relation is found for 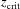: 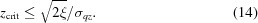
Below 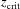, 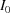 is a good approximation for the measured intensity. Above 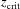, the resolution has to be taken into account. For 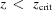, variations in the envelope of the measured intensity can be directly related to microstructural changes of the sample. Above 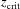, only deviations larger than the resolution effect [equation (12)] can be unequivocally attributed to the sample. For our VC_1–*x*_/a-C coatings, 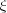 = 0.05 is a reasonable value, taking into account the experimental error. The experimental value of 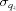 = 0.002 Å^−1^ results in 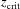 ≃ 16 nm. For a typical 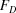 = 0.22 nm s^−1^ this corresponds to a deposition time of 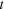 = 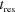 = 68 s. Since the typical coating thickness for hard coating materials is of the order of micrometres, the intensity is significantly modified by the resolution almost throughout the entire deposition time. Therefore we included resolution effects in the analysis of the *in situ* data as shown in the next section.

## 5. Results   

### 5.1. Deposition of a VC_1–*x*_/a-C thin film at different DC power at low pressure   

The applied DC power can affect the number of particles sputtered at the target, as well as their initial energetic and angular distribution (Waits, 1978 ▶). To understand the influence of the DC power on the thin film formation of VC_1–*x*_/a-C during the sputtering from a compound target, *stationary* *in situ* measurements during the growth of the sample P-step were performed.

**Figure 5:**
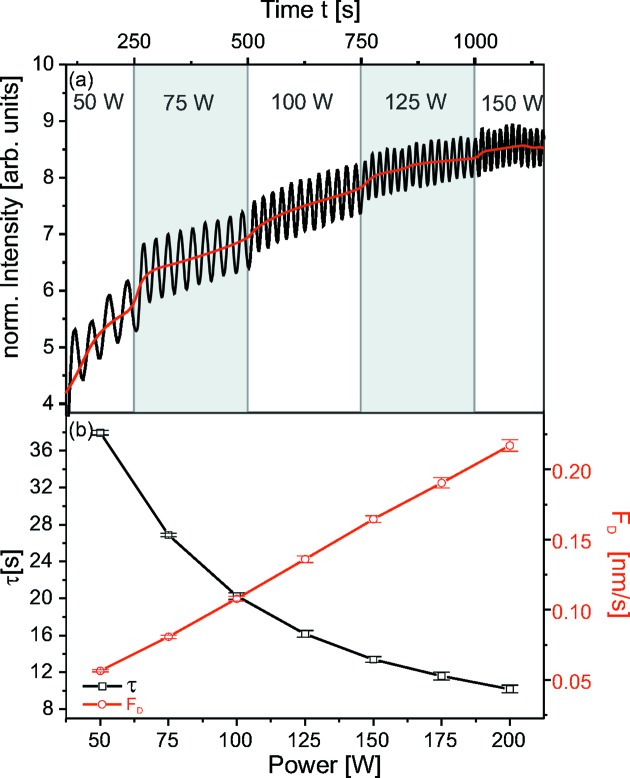
(*a*) *Stationary* *in situ* measurement during the first five power steps. The mean intensity is marked by a red line. (*b*) Oscillation period τ for different DC powers (black squares) and calculated 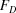 in nm s^−1^ from oscillation period (red circles).

Fig. 5(*a*) ▶ presents the intensity of the reflected beam during the first five power steps of 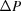 = 25 W. While the power is kept constant, the period is constant. When the power is increased by 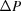, the period decreases stepwise with the increased power. For each power step, 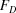 was calculated from the period of the growth oscillation using equation (8). Fig. 5(*b*) ▶ summarizes the results. The period length (black squares) was determined by fitting a 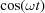 function [equation (7)]. The fitting error of τ is smaller than 2%. 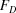 (red circles) is proportional to the applied DC power.

The mean intensity, marked by the red line in Fig. 5(*a*) ▶, is continuously increasing at constant power. When the power is increased, small jumps in mean intensity are visible. Changes of the mean intensity can only be related to variations in electron density and roughness of the sample.

**Figure 6:**
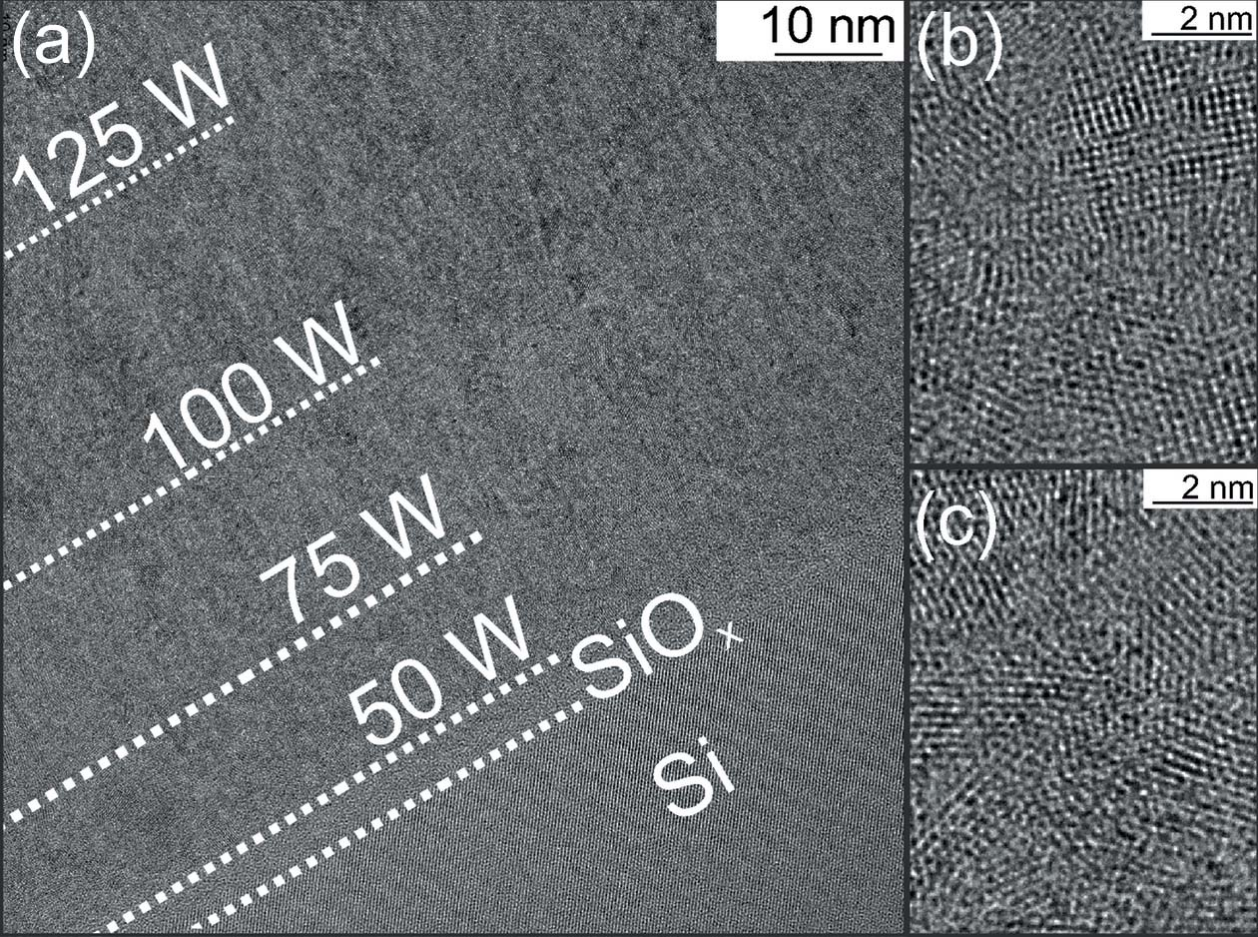
(*a*) HRTEM cross-section image of the thin film. Regions at DC power of 50 W (*b*) and 200 W (*c*) are highlighted for better comparison.

The intensity was simulated based on the Parratt algorithm, assuming only electron density changes. For achieving a satisfactory agreement with the experimental data, density changes larger than 50% between the coatings at 50 W and 200 W were needed. This is not consistent with the HRTEM images of the film presented in Fig. 6(*a*) ▶. Highlighted regions of the thin film at 50 W (*b*) and 200 W (*c*) show no major changes in the microstructure. Therefore the increase in the mean intensity must be dominated by a smoothening of the coating. The roughness decreases continuously at constant power, but reduces almost instantly when the power is increased. These sudden changes are only accessible during *in situ* experiments. With increasing thickness, usually an increase in roughness is expected (Thornton, 1977 ▶; Shaha *et al.*, 2010 ▶). The reason for the contrary result found here is discussed in more detail in the next section.

Since no significant changes in electron density were found, 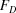 is dominated by the increasing deposition rate due to higher DC power and not by microstructural changes. This is in good agreement with the linear relation between power and deposition rate found for single-element targets (Waits, 1978 ▶). This linear behaviour shows also that in our studied range the initial angular and energetic distribution is independent of the power. This was confirmed by *TRIDYN* simulations performed for different energies of the impinging Ar ions (adapted to the DC powers).

### 5.2. Deposition of VC_1–*x*_/a-C thin films at different working gas pressures   

The angular and energetic distribution of the sputtered particles depends also on their transport through the gas phase. A simple way to influence the microstructure formation is the variation of the working gas pressure. In the following we study the microstructure formation under *steady growth conditions*, where the pressure as well as the applied power are constant (excluding the more complex plasma ignition period). Samples were deposited under low-pressure (Lp) and high-pressure conditions (HP).

**Figure 7:**
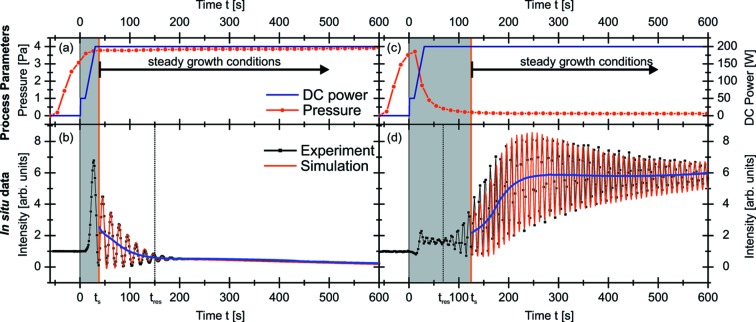
Selected process parameters [(*a*) and (*c*)] and *stationary* *in situ* measurements [(*c*) and (*d*), black dotted lines] at high and low working gas pressure, respectively. The mean value of the intensity is marked by a purple line. Simulations are presented with red lines. The orange line marks the beginning of steady growth conditions.

Fig. 7 ▶ shows the *in situ* X-ray measurements, their simulations and selected process parameters for both samples. The left column corresponds to HP, the right column to Lp. The process parameters of both samples are presented in the upper row [(*a*) and (*c*)], the *in situ* X-ray measurements and their simulations in the lower row [(*b*) and (*d*)]. The main process parameters are the DC power (blue line) and the working gas pressure (red dots). In the case of HP, the pressure is slightly increasing after plasma ignition at 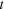 = 0 s. *Steady growth conditions* are reached at 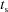 ≃ 37 s, marked by an orange line. For Lp, the pressure is decreasing after plasma ignition, and *steady growth conditions* are reached at 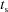 ≃ 122 s. The initial growth region with 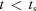 is shaded in grey. The experimental X-ray data are represented by black dotted lines, the mean intensity by blue lines. Their simulations (red lines) are shown for the steady growth region. Both *in situ* measurements show the typical growth oscillations, but it is instantly visible that the pressure has a strong influence on the structure formation. In the *steady growth regime*, both *in situ* curves oscillate with constant period. At high pressure, 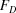 = 0.132 ± 0.003 nm s^−1^ was estimated using equation (8). At low pressure, the estimated 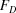 = 0.215 ± 0.003 nm s^−1^ is larger by almost a factor of two than at high pressure. This difference is related to a higher collision rate at higher pressure, which will be discussed later.

As shown in Fig. 7 ▶, the envelope of the growth oscillations is very sensitive to pressure-induced changes of the microstructure. At high pressure, the amplitude decreases and is completely damped at 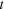 > 200 s. At low pressure, the amplitude increases up to 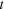 = 200 s, then decreases slowly. For HP, 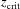 is reached at 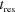 ≃ 150 s, assuming a constant 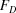 in the initial growth phase. Therefore the damping of the growth oscillations for 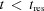 must be related to roughness changes. For Lp, 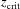 is reached at 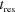 = 68 s. Since 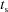 ≃ 122 s > 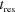, the damping of the amplitude in the later growth region is strongly influenced by resolution effects even if roughness effects cannot be excluded.

The details of the time-dependent microstructure formation were determined by fitting a simple model to the experimental data (§4). The coating was assumed to be a layer with increasing thickness *D*(*t*), where roughness and electron density changes only happen at the growth front. These parameters were optimized, using the estimated 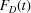, as well as the average δ determined from the refractive index *n* = 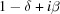 of the post-growth XRR measurements as start values.

**Figure 8:**
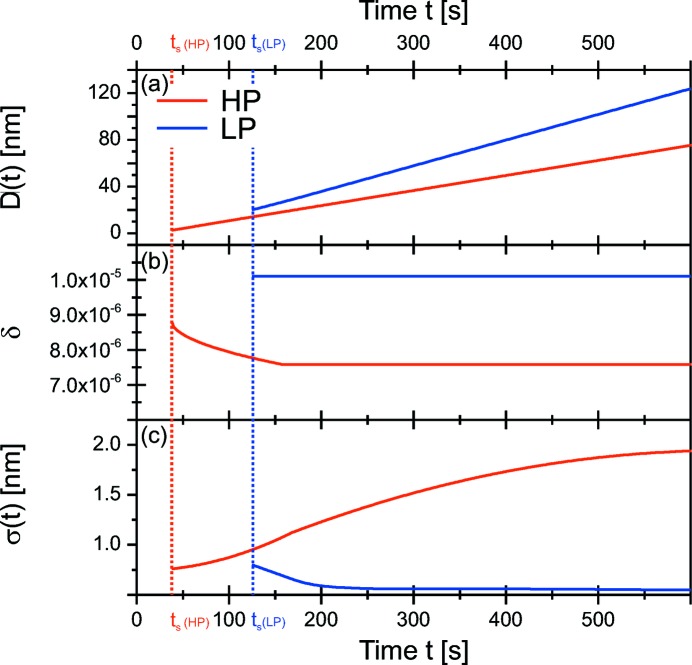
Simulation input for the *stationary* measurements: (*a*) thickness *D*(*t*), (*b*) δ(*t*), (*c*) σ(*t*). *t*_s_ of both samples are indicated by dotted lines.

Within the model, both 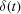 and 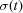 influence the amplitude and the mean value of the intensity in a characteristic way. In our case, 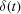 and 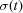 are continuous and vary only slowly compared with the oscillation period. Therefore, 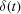, 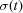 and *D*(*t*) can be determined with an estimated precision of around 1%. The results are presented in Fig. 8 ▶. As estimated, the thickness *D*(*t*) of both films is increasing almost linearly with time. However, small deviations in 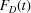 at high pressure of 5% could only be revealed by the fitting.

For Lp, the expected decrease in 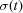 of the film up to 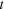 = 200 s was confirmed. Although the amplitude for 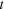 > 200 s is mainly influenced by the resolution, a further slight smoothening was determined by the fitting. For HP, a strong increase in roughness was found even after the complete damping of the oscillations.

The fitting revealed also a decrease in the mean electron density for 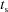 < *t* < 130 s, corresponding to 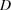 < 14 nm. For Lp the constant density was observed for 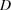 > 20 nm. This might be explained by the influence of the substrate on the growth process for lower thicknesses.

**Figure 9:**
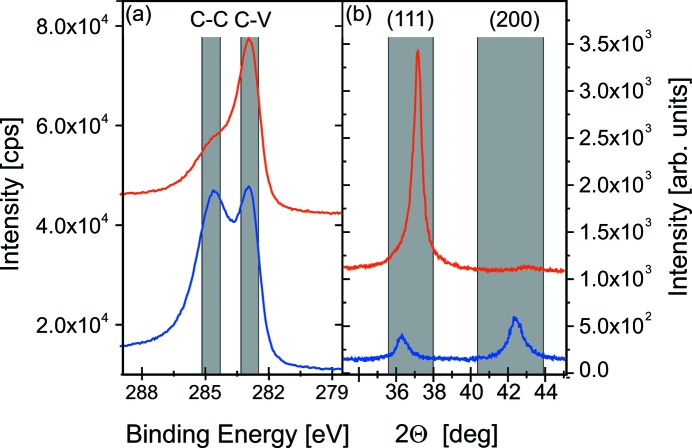
(*a*) XPS spectra of the C 1*s* peak performed after deposition of the sample grown at high pressure (upper curve) and low pressure (lower curve). (*b*) Θ–2Θ scans performed after deposition of the sample grown at high pressure (upper curve) and low pressure (lower curve). The curves in (*a*) and (*b*) are vertically shifted for better comparison.

To understand these differences in thin film formation at the different working gas pressures, simulations of the sputtering process were performed (§2). The results are presented in Table 1 ▶. Consistent with the experiment, the calculated flux 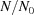 decreases with increasing pressure. At high pressure, the energy-dependent mean free path (MFP) is strongly reduced and more particles are scattered to the side (Abadias *et al.*, 2009 ▶). The simulations predict a change in chemical composition from a C/V ratio of 0.9 at low pressure to C/V = 1.1 at high pressure. For experimental verification, XPS measurements were performed (Fig. 9*a* ▶).

For both pressures, the measurements revealed a splitting of the C 1*s* peak. The peak at 284.5 ± 0.5 eV can be attributed to C—C bonding, at 282.9 ± 0.5 eV to C—V bonding (Liao *et al.*, 2005 ▶; Teghil *et al.*, 2009 ▶), as expected for f.c.c. VC_1–*x*_/a-C composite material. The quantitative analysis of the spectra confirms qualitatively the *SIMTRA* results with C/V = 1.3 (Lp) and C/V = 0.9 (HP). The changes in composition at different pressure can be explained by the significant differences in the mass of the two elements. The lighter C atoms are sputtered off-axis with a higher probability than the heavier V atoms. With increasing pressure, more collisions take place and the number of C atoms scattered away from the target increases. Similar results for compound targets with large mass differences have been reported in the literature (Liao *et al.*, 2004 ▶; Eklund *et al.*, 2007 ▶; Neidhardt *et al.*, 2008 ▶; Mraz *et al.*, 2013 ▶).

XRD measurements were performed to investigate the influence of the working gas pressure on the texture formation. For both samples the typical rock salt structure was found. In Fig. 9 ▶, the main peaks are indicated by grey boxes. HP exhibits a strong (111)-texture, while Lp is weakly textured.

For TiC, it was found that a low amount of C leads to a fibre texture, while films with a higher C content are less well oriented (Stüber *et al.*, 2002 ▶; Martinez-Martinez *et al.*, 2009 ▶; El Mel *et al.*, 2010 ▶; Samuelsson *et al.*, 2012 ▶). This is explained by the suppression of grain growth due to C. VC exhibits a similar texture change with composition. This indicates that the same growth mechanisms are relevant.

The differences in the texture driven by compositional changes explain well the variations in the roughness with time. At high pressure, a fibre texture is evolving. This leads typically to a higher roughness of the films due to the strong faceting (Zhang *et al.*, 2005 ▶). For Lp, the initial high pressure leads also to a roughening of the film. With decreasing pressure, the content of a-C increases. Due to C-induced renucleation, a nanocomposite film grows with small grains, thus resulting in a smooth film.

## 6. Summary   

In this manuscript we showed that *stationary* measurements are a powerful tool for monitoring the thin film formation, independent of their crystalline structure. The presented theoretical approach gives a qualitative understanding of the envelope and the period of the growth oscillations. This allows the online-interpretation of the measured intensity in terms of growth velocity, roughness and electron density changes. Furthermore, our method allows the quantitative analysis of these parameters. A direct extraction of the parameters from the *in situ* data is possible for simple growth processes, while more complex systems can be evaluated using growth models.

We demonstrated our approach by monitoring the film formation of the composite material VC. Instant as well as slowly varying temporal variations in the microstructure formation were revealed. The *in situ* XRR measurements showed that the DC power affects mainly the deposition rate. The working gas pressure does not only influence the deposition rate but changes significantly the time-dependent development of the roughness and electron density of the coatings. For the interpretation of the observed changes during the film formation, the *in situ* data were supported by results of complementary methods and simulations. Using this combined approach, the chemical composition was identified as the main driving force on the microstructure formation at different working pressures. Due to the high intensity of the reflected beam, *in situ* XRR measurements can be performed using conventional X-ray laboratory sources. However, synchrotron radiation gives access to faster processes and to the lateral correlation by monitoring simultaneously the diffuse scattering.

## Figures and Tables

**Table 1:** The average energy *E*_sp_ of the sputtered particles was determined by *TRIDYN* simulations. From this, the energy-dependent mean free path at *p* = 0.2Pa and *p* = 4Pa was calculated. The fraction of the sputtered particles arriving on the substrate *N*/*N*_0_ was simulated using *SIMTRA*

		MFP (cm)	MFP (cm)	*N*/*N*_0_ (%)	*N*/*N*_0_ (%)
Element	*E*_sp_ (eV)	(0.2Pa)	(4Pa)	(0.2Pa)	(4Pa)
C	12.26	20.11	1.01	0.0155	0.0026
V	8.08	18.44	0.92	0.0142	0.0027
